# Municipality-Level Checklist to Promote Parental Behaviors Related to Prevention of Unintentional Injury in Young Children: A Multilevel Analysis of National Data

**DOI:** 10.2188/jea.JE20190079

**Published:** 2020-10-05

**Authors:** Makiko Sampei, Tsuguhiko Kato, Aurelie Piedvache, Naho Morisaki, Junko Saito, Yuka Akiyama, Ryoji Shinohara, Zentaro Yamagata, Kevin Y. Urayama, Naoki Kondo

**Affiliations:** 1 Department of Social Medicine, National Center for Child Health and Development, Tokyo, Japan; 2 Division of Prevention, Center for Public Health Sciences, National Cancer Center, Tokyo, Japan; 3 Department of Health Education and Health Sociology, Department of Health and Social Behavior, School of Public Health, The University of Tokyo, Tokyo, Japan; 4 Department of Health Sciences, School of Medicine. University of Yamanashi, Yamanashi, Japan; 5 Center for Birth Cohort Studies Graduate School of Interdisciplinary Research, University of Yamanashi, Yamanashi, Japan; 6 Graduate School of Public Health, St. Luke's International University, Tokyo, Japan

**Keywords:** unintentional injury, parental safety behavior, Healthy Parents and Children 21, population, multilevel analysis

## Abstract

**Background:**

Unintentional injury is a major cause of morbidity and mortality among young children in developed countries. In this national study, we examined the role of municipality-level safety checklist implementation for reducing risky child-safety-related parental behaviors.

**Methods:**

Nationwide data were collected to evaluate the impact of the *Healthy Parents and Children 21* initiative of the Japanese government. Questionnaires related to safety checklist implementation were administered to a random sample of municipal offices and to parents at the child’s routine 1.5-year health exam on parental behaviors related to child safety. Adjusting for municipality and individual-level variables, multilevel analysis was used to examine the relationship between municipality checklist implementation (4-month health exam) and six child-safety-related parental behaviors at the 1.5-year health exam.

**Results:**

Families (*n* = 23,394) across 371 municipalities in Japan were included in this study; 5.6% of municipalities implemented a child safety intervention. Living in a municipality with a checklist intervention was associated with reduction in certain risk behaviors (not keeping tobacco/ashtray and candy out of the reach of infants, not using a car seat, not having a lock on bathing room door). However, after additionally taking into account municipality-level residual effects, only the “tobacco” behavior showed association with municipality of residence (Interval odds ratio, 0.25–0.94) and others were weak in the context of other potential municipality-level influences.

**Conclusions:**

A municipality-level intervention taking a checklist-based approach at the 4-month health exam in Japan appears to promote certain child safety behaviors in parents with children around 1.5 years of age.

## INTRODUCTION

Injuries are a major cause of death among children in developed countries, accounting for up to about 40% of all deaths during childhood.^1^ In Japan, unintentional injury is a major cause of mortality among children aged 0 to14 years, leading to about 300 deaths each year.^2^ During the past decade, advancements in medical technology have led to reductions in mortality due to unintentional injuries in Japan, from 525 deaths in 2000 to 157 deaths in 2016 among children aged 0–4.^2^ However, the number of ambulatory visits by children 0 to 14 years old has remained relatively constant at approximately 46,900 (0.3%) and 42,100 (0.3%) in 2005 and 2014, respectively.^3^

Most of the injuries in children under 2 years old occur at home, so providing public health measures that contribute to maintaining a safe home environment should be effective.^4^^–^^6^ However the results of studies evaluating policy-level approaches to prevent unintentional injuries by intervening on parental behavior have been inconsistent.^7^^–^^10^ One reason for the inconsistent findings across previous studies may be due to the diversity of intervention content and quality of implementation of the intervention.^8^

A checklist approach may be suitable for standardized and effective delivery of injury prevention information across a broad population. The checklist approach has been used in various settings, including medical safety (eg, surgical procedures) and the airline industry.^11^ As for its use in injury prevention, it can serve as a conventional tool for educating parents but also serves as a means of social support provided by municipality health professionals.^12^^–^^14^ It may contribute to an effective “choice architecture” that offers options for safer and healthier daily living by providing childbearing guardians with structure for complex and overwhelming daily tasks.^15^^–^^17^

Previous studies have demonstrated potential utility of a checklist approach to injury prevention. In one study, which implemented a county-based intervention that included a checklist exhibiting safety equipment and products, the incidence of injury in children and the elderly was significantly reduced compared to a second county that did not take this approach.^18^ In another study, information, including age-specific safety behaviors checklists, was given to parents of children 0–14 years old. During an evaluation period of 8 years, the number of children experiencing injury appeared to decreased but the difference was not significant.^19^ Due to limited studies conducted to date, it is still inconclusive as to the potential utility of a checklist-based approach to injury prevention in young children.

Over the last several years, the Japanese government has encouraged municipalities to implement regional policy-based approaches to educating and informing families with young children about childhood injuries, which has included an option to use the checklist. Based on national guidelines, each municipality determines which approach to adapt, with one option being to administer a standardized checklist. As a fairly large number of municipalities accepted this guideline, this created a circumstance suitable for investigating potential effects of a checklist program.

In the current study, we evaluated the relationship between living in a municipality where an infant safety checklist was administered at the 4-month health examination and child-safety-related parental behaviors. Using multilevel modeling to account for both individual-level and regional characteristics, we hypothesized that parents who live in a municipality that implemented a safety checklist to parents within their program for the prevention of unintentional injuries would have a decreased likelihood of engaging in unsafe behaviors known to be associated with unintentional injuries in young children.

## METHODS

### Study setting

**Figure 1.**
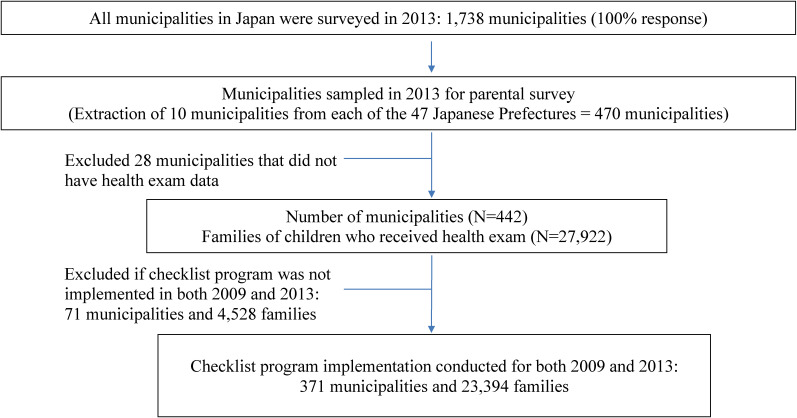
Flow diagram of the number of municipalities and families included in this study. While surveys were conducted over multiple years, this study focused on municipalities surveyed in 2013 and parental surveys administered at a child’s 1.5 month old health exam in 2013.

This study is a cross-sectional design using national survey data across multiple years. The data came from a research project led by Yamagata and colleagues^20^^,^^21^ to evaluate the effect of the *Healthy Parents and Children 21*,^22^ a governmental initiative implemented by the Japanese Ministry of Health, Labour and Welfare in 2001 (eAppendix 1). The data used in the current study were obtained from two types of surveys: one targeted municipal officials regarding the implementation status of projects (referred to as municipal survey) in 2009 and 2013, and the other was administered to parents at the time of the child’s routine health exam (referred to as parental survey) in 2013. While the municipal survey was administered across all 1,738 municipalities in Japan, the parental survey was administered by only a subset of municipalities selected randomly after stratifying by population size (eAppendix 1). Within each prefecture (large administrative divisions of Japan), municipalities were ranked by ascending population size and were divided at quartile cut points. Two municipalities were selected at random from each of the lower two quartiles, and three each from the upper two quartiles, resulting in 10 municipalities in each of the 47 prefectures (Figure 1).^20^^,^^21^ We excluded municipalities that did not administer a parental survey at the 1.5-year health exam in 2013 (*n* = 28), and those that did not report the same checklist implementation status in 2009 and 2013 (*n* = 71). The municipal survey was not conducted in 2012; therefore, this latter exclusion criteria was necessary to ensure “exposure” status assignment of the checklist program at the time of the 4-month health exam that occurred in 2012, which was the exam prior to outcome assessment at the 1.5-year exam in 2013 (time of parental survey for behavioral assessment) (Figure 1 and eAppendix 1).

Parental surveys administered at the 1.5-year health examination in 2013 were used to obtain individual-level information on child-safety-related parental behaviors (dependent variable or “outcome”) and other parental and family characteristics. As shown in Figure 1, 371 municipalities and 23,394 parental data were analyzed.

### Infant safety checklist

The Ministry of Health, Labour and Welfare has recommended using an “Infant Safety Checklist” (from here on referred to as the “checklist”) with age-specificity and standardization.^23^ Each municipality received guidelines for the prevention of unintentional injuries in children as part of the *Healthy Parents and Children 21* initiative. In the checklist, parents perform a self-assessment based on 20 items related to parental behaviors that have the potential to prevent unintentional child injuries. For example, in Japan, it is common for water to be left in the bathtub for reuse by multiple individuals and presents a drowning risk for young children (eFigure 1).

In the municipal survey, municipalities were asked about whether they had implemented some programmatic measure in the setting of the child’s health exam that addressed the prevention of unintentional injuries in children. The response options consisted of implementation of checklists, brochure distribution, use of bulletin boards, videos, individual guidance, group guidance, and other interventions. Among them, we used data from the 2009 and 2013 municipal surveys for information on the implementation status of a checklist.

### Outcomes

Child-safety-related parental behaviors, obtained from the parental survey, were the outcome variables of interest. Because suffocation, traffic accidents, and drowning are the major causes of unintentional injuries among children in Japan,^3^^,^^24^ the analyses focused on these three themes which were represented by six specific questions. The first theme was related to risk of suffocation and included the following questions: 1-a) Do you always keep tobacco or ashtrays out of your child’s reach? (referred to as “tobacco”); 1-b) Do you keep peanuts and candy out of your child’s reach? (referred to as “candy”); and 1-c) Do you place pills, cosmetics, detergents, etc out of your child’s reach? (referred to as “pills/detergents”). Related to risk of traffic accident, the following single question was included: 2) When riding in a car with your child, have you installed a child car seat in the back seat? (referred to as “no child car seat”). Questions related to risk of drowning included the following two questions: 3-a) Do you try not to leave water in the bathtub? (referred to as “undrained bathwater”); and 3-b) Is there any lock on the door of the bathing room that stops children from opening the door? (referred to as “no bathing room lock”). The number of responses to each question may have varied since certain risk behaviors may not have been applicable to some parents. For instance, the parents who did not smoke were excluded from the analysis of the “tobacco” outcome.

### Covariates

Individual-level characteristics, which were obtained from parental survey data, included birth order, child’s sex, maternal age, maternal occupation, economic status, family/friend support, and having a family physician. Municipality-level characteristics included population density, the percent of children aged 0 to 3 years, the percent unemployed, and mean taxable income, which were included as potential confounders (eAppendix 1).

As this is a secondary analysis of an existing data set with no access to personal identifiers, the requirement for informed consent was waived. Ethics approval was obtained from the institutional review boards of the University of Yamanashi and the National Center for Child Health and Development, Japan.

### Statistical analysis

First, we described the prevalence of each risk behavior in families, overall and grouped by checklist implementation status, and showed the median prevalence across municipalities and interquartile range (IQR). Differences in municipality characteristics by checklist implementation status were evaluated using the Wilcoxon-Mann-Whitney test. Next, we used a two-level logistic regression model with parental variables considered at level 1 and municipality variables at level 2 to examine the association between each child safety related parental behavior and checklist status. As the latter is fixed within municipality, the 80% interval odds ratio (IOR) including municipality level residual variation was computed to accurately interpret the odds ratio.^25^^,^^26^ In addition, the magnitude of the municipality effect quantified by the median odds ratio (MOR), and the effect of checklist status evaluated by the proportional change in cluster variance (PCV) were estimated as a measure of inter-municipality variations. The variance partition coefficient (VPC) was calculated to assess the proportion of variation explained by municipalities.

Four regression models were pursued. The null model (model 0) was used as the reference to estimate the percent change in variance across municipalities. Model 1 included only individual variables. In model 2, municipality characteristics were added and the final model (model 3) included all variables from previous models in addition to the checklist status variable. Among factors that influence risk behaviors, family characteristics and social factors play an important role. It is, therefore, important to show how these factors may explain municipality differences independently of any program of prevention. Hence, we made the choice of performing a four-step multilevel analysis accounting for individual and municipality factors successively.

Stratified analyses were performed by birth order and population size of the municipality. Birth order was considered because it is generally believed that care practices may be different between firstborns and non-firstborns. We stratified by municipality population size because municipalities with small population sizes may have potentially different program implementation settings. A two-sided *P*-value of less than 0.05 was considered statistically significant in all analyses. Statistical analyses were performed using STATA/SE 13.1 software (Stata Corp, College Station, TX, USA).

## RESULTS

The median prevalence of child safety related parental behaviors (outcome) across the 371 municipalities are shown in Table 1 and ranged from as low as 3% for “tobacco” to 61% for “no bathing room lock”. Of the six behavior outcomes evaluated, bivariate multilevel logistic regression of municipality checklist status showed crude associations with “tobacco” (*P* = 0.02), “candy” (*P* < 0.01), “no child car seat” (*P* = 0.06), and “no lock on bathing room” (*P* = 0.02). Among the municipality-level variables evaluated, regions implementing a checklist were observed to have higher proportions of children aged 0–3 years (*P* = 0.01) (Table 2).

**Table 1.** Frequencies of parental child safety related behaviors by municipalities providing an infant safety checklist program

	Number of families	Families living in a checklist municipality	Families living in non-checklist municipality	Number of municipalities (*N* = 371)	Municipalities providing a checklist program (*N* = 16)	Municipalities not providing a checklist program (*N* = 355)
	*n*/*N* (%)	*n*/*N* (%)	*n*/*N* (%)	Median prevalence across municipalities [IQR]	Median prevalence across municipalities [IQR]	Median prevalence across municipalities [IQR]
Risk of suffocation						
Tobacco	435/13,103 (3%)	10/629 (2%)	425/12,474 (3%)	2.2 [0–4.9]	0 [0–1.9]	2.4 [0–5.2]
Candy	1458/22,769 (6%)	40/1108 (4%)	1418/21,661 (7%)	6.3 [3.9–8.9]	3.3 [2.4–5.5]	6.6 [4.0–9.1]
Pills/detergents	4286/22,769 (19%)	188/1111 (17%)	4098/21,658 (19%)	18.8 [14.0–23.4]	18.6 [8.7–24.5]	18.8 [14.0–23.4]
Risk of traffic accident						
No child car seat	2221/21,340 (10%)	85/1054 (8%)	2136/20,286 (11%)	10.5 [6.5–15.4]	8.0 [5.6–11.9]	10.5 [6.5–15.4]
Risk of drowning						
Undrained bathwater	5640/22,143 (25%)	224/1085 (21%)	5416/21,058 (26%)	24.0 [17.0–33.3]	21.5 [16.8–33.9]	24.1 [17.2–33.3]
No lock on bathing room	13,656/22,239 (61%)	624/1088 (57%)	13,032/21,151 (62%)	62.5 [56.9–68.1]	58.7 [49.5–63.0]	62.5 [57.1–68.4]

IQR = interquartile range; *n* = number with the outcome behavior; *N* = number of total samples.

**Table 2.** Municipal characteristics by implementation status of a infant safety checklist program

	Municipality checklist program status		*P* value^b^

	Yes (*N* = 16)	No (*N* = 355)	
	
	Median (IQR)	Median (IQR)	
Population density^a^	9.0 (5.6–17.6)	6.6 (3.5–13.5)	0.25
Percent aged 0–3 years	3.5 (3.2–3.7)	3.1 (2.6–3.5)	0.01
Percent unemployed	6.3 (5.0–7.2)	6.3 (5.2–7.2)	0.82
2013 taxable income, per ten billion yen	6.2 (2.4–10.0)	4.3 (1.8–13.2)	0.89

IQR, interquartile range.

^a^Dividing the number of the people in the municipality by habitable areas in which people can live; the unit is person/hectare.

^b^Wilcoxon-Mann-Whitney test.

Table 3 presents the results of the multivariate multilevel analysis for the four parental behaviors that showed statistically significant differences by municipality checklist status in the bivariate analysis. The empty model, which included only the municipality-level random intercept, showed highest levels of baseline variation for “tobacco” (VPC = 4.9%) and “no child car seat” (VPC = 4.6%). Sequential model expansion for “tobacco” showed increasing proportions of variation being explained by individual-level variables (mode 1, PCV = 6%), municipal-level variables (model 2, PCV = 18%), and the addition of the checklist variable (model 3, PCV = 24%). Increases were also observed for “no child car seat” in model 1 (PCV = 25%), model 2 (PCV = 44%), and model 3 (PCV = 50%). Notable increases in PCV were not observed for the other outcome behaviors when checklist was added in model 3, suggesting its minimal role in explaining additional variation. The importance of municipality-level characteristics in explaining differences in individual-level “tobacco” and “no child car seat” behaviors across municipalities is supported by the elevated MORs (1.41 and 1.31, respectively).

**Table 3.** Multilevel logistic regression analysis evaluating the relation between infant safety checklist and parental behaviors

	Empty model	Individual level^a^	Individual and municipal level^b^	Individual and municipal level checking list^c^
	(Model 0)	(Model 1)	(Model 2)	(Model 3)
**Tobacco**				
*Measures of association*				
Checklist, OR (95% CI)^†^				0.49 (0.25–0.95)
IOR-80%				(0.25–0.94)
*Measures of variation*				
Variance	0.17	0.16	0.14	0.13
VPC	4.9%	4.6%	4.1%	3.8%
PCV	ref	6%	18%	24%
MOR	1.48	1.46	1.43	1.41
**Candy**				
*Measures of association*				
Checklist, OR (95% CI)^†^				0.54 (0.39–0.75)
IOR-80%				(0.54–0.54)^d^
*Measures of variation*				
Variance	0.02	0.01	0.00	0.00
VPC	0.6%	0.3%	0.0%	0.0%
PCV	ref	50%	100%	100%
MOR	1.14	1.10	1.00	1.00
**No child car seat**				
*Measures of association*				
Checklist, OR (95% CI)^†^				0.72 (0.54–0.97)
IOR-80%				(0.43–1.22)
*Measures of variation*				
Variance	0.16	0.12	0.09	0.08
VPC	4.6%	3.5%	2.7%	2.4%
PCV	ref	25%	44%	50%
MOR	1.46	1.39	1.33	1.31
**No lock on bathing room**				
*Measures of association*				
Checklist, OR (95% CI)^†^				0.85 (0.73–0.99)
IOR-80%				(0.66–1.10)
*Measures of variation*				
Variance	0.03	0.02	0.02	0.02
VPC	0.9%	0.6%	0.6%	0.6%
PCV	ref	33%	33%	33%
MOR	1.18	1.14	1.14	1.14

CI, confidence interval; MOR, median odds ratio; OR, odds ratio; PCV^e^, proportional change in cluster variation; ref, reference; VPC^f^, variance partition coefficient.

^a^Individual level variables included maternal age (year), birth order, child’s sex, maternal occupation, self-assessed economic status, persons to consult, have family physician.

^b^Municipality level variables included population density, percent of population aged 0–3 years, unemployment rate, taxable income 2013.

^c^Adjusted for individual and municipality level characteristics (see eTable 1, eTable 2, eTable 4, and eTable 6 for full results).

^d^As the variance is null, IOR is the same as the odds ratio. $\text{IOR}=[\text{exp}(\beta -\sqrt{2*\sigma^{2}}\varphi^{-1}(0.10);\exp(\beta +\sqrt{2*\sigma^{2}}\varphi^{-1}(0.90)]$.

^e^To assess how much of the cluster-level variance is explained by differences in the covariables. $\text{PCV }(\%)=\frac{\sigma^{0}-\sigma^{1}}{\sigma^{1}}\times 100$.

^f^To assess the proportion of the total observed individual and municipality variation in the outcome that is attributable to between-cluster variation. $\text{VPC}=\frac{\sigma^{2}}{\sigma^{2}+\frac{\pi^{2}}{3}}$.

Statistically significant municipality-level fixed effect OR (model 3) for checklist was observed for “tobacco” (0.49; 95% confidence interval [CI], 0.25–0.95), “candy” (0.54; 95% CI, 0.39–0.75), “no child in car seat” (0.72; 95% CI, 0.54–0.97), and “no lock on bathing room” (0.85; 95% CI, 0.73–0.99), indicating an association. However, examination of the IOR showed a marked effect of checklist only for the “tobacco” behavior (IOR = 0.25–0.94) when placed in the context of the remaining residual municipality-level heterogeneity. The interpretation of the IOR is that if we select randomly two parents with identical covariates but, one living in a municipality where a checklist program is administered and the other living in a municipality where it is not, the interval odds ratio associated with “tobacco” lies between 0.25 and 0.94. The “candy” behavior also showed a notable IOR, but should be interpreted with caution as variation in this behavior across municipalities was minimal with a large proportion of the variation being explained by individual-level characteristics. IOR associated with the other behaviors all included 1.0, providing weak evidence for a role of the checklist relative to other possible municipality-level explanations (eTable 1, eTable 2, eTable 3, eTable 4, eTable 5, and eTable 6).

Sensitivity analyses excluding small municipalities that contributed fewer than 10 people showed similar results (data not shown). Also, in contrast to our hypothesis, no strong evidence of heterogeneous effects of checklist on parental behaviors was observed by strata of birth order and population size (*P* for interaction >0.1). A reduction in statistical power resulted in stratum-specific results which lost statistical significance; however, the risk estimates remained consistent (eTable 7).

## DISCUSSION

Using national surveys administered both to municipality officials and families with young children visiting for a routine health exam, this large-scale analysis examined whether a municipality-level checklist intervention was associated with child-safety-related parental behaviors, particularly related to children’s risks of suffocation, traffic accident, and drowning. After accounting for both potential municipality-level and individual-level confounders, we observed that municipality implementation of checklist was associated with parental behavior, specifically related to keeping tobacco/ashtray out of the reach of infants. Regarding the other parental behaviors such as using a child car seat and having a lock on the bathing room door, although an association was detected, we observed the checklist relationship to be weak in the context of other potential municipality-level influences.

Variations in the parental behavior of keeping tobacco/ashtray out of the reach of infants across municipalities had a baseline municipality variance of 0.17. This translates to 4.9% of the residual variation in the parental behavior being explained by systematic difference between municipalities, while the remaining 95.1% is due to between-individual differences. Compared to the baseline model, successively adding individual-level, municipality-level, then checklist status to the model resulted in gradual increases in the proportion of between-municipality variation in parental behavior explained (24% in the final model). The adjusted OR in the final model showed a marked association between checklist and tobacco-related parental behavior (OR 0.49; 95% CI, 0.25–0.95), with an IOR-80 that excluded unity (0.25–0.94). In summary, the results of the systematic approach taken in this analysis to examine the sources of variation suggests that the effect of checklist status is notable relative to the underlying municipality-level effect.

The observed association with keeping tobacco/ashtray out of the reach of infants may be explained by persistent public health initiatives over the years, the harmful effects of tobacco smoke is now clearly acknowledged by the general population and parents perceive it as a realistic threat to their children. A simple reminder through the checklist may have encouraged parents who smoke to alter this behavior more than other behaviors, which they may have perceived as less of a risk.

Most previous studies reporting on municipality-level intervention and childhood injuries applied ecological designs and targeted serious injuries among hospitalized children. Within this setting, studies conducted in the United States have shown that municipality interventions, including education through mass media, have no effect on reducing deaths and hospitalizations due to poisoning among children under the age of 5 years.^27^^,^^28^ Moreover, in a study where the municipality carried out multiple interventions, including a checklist program, no association with injuries was observed among reports studying children of a broad age range of 0 to 14 years.^19^

There are several potential reasons for these differences in results. First, previous studies included a wider age range of children, despite mechanisms of injury likely varying by age group. Second, the studies implemented a multi-intervention approach that may have obscured any specific effect of a checklist strategy. Third, most previous studies were ecological in design, which may be considered less rigorous than the multilevel analytical approach we pursued to adjust for potential confounding originating from both individual- and municipality-level characteristics. Finally, in Japan, the variation in the style of living quarters may be smaller than those in many western countries, allowing for straightforward implementation of preventive behaviors based on checklist specifications. In addition, we considered that first-time parents may have different tendencies than experienced parents and that the implementation quality of the checklist program could be affected by the population load of the municipality. We did not observe clear evidence for differential effects across strata, but we acknowledge that our ability to perform a thorough evaluation of the hypothesis was affected by weakened statistical power.

This study had several strengths. First, the use of a nationally administered survey provided us the benefit of sufficient statistical power for the primary analysis and the ability to generalize the results to the Japanese general population, as well as serving as a model example for potential programs in other countries. Second, unique to this study was our use of the multilevel analysis approach that allowed for the appropriate adjustment of individual correlations within the municipality giving us added confidence in the interpretation of the results. Previous studies mostly utilized ecological evaluation designs or a demographically matched comparison community^29^ without employing multilevel analytical approaches.

This study had some limitations. First the checklist implementation status of the municipality in 2012 was assumed based on the status in 2009 and 2013. While the likelihood of misclassification is low based on these criteria, the association may be underestimated due to some non-differential misclassification. Second, parental behaviors were self-reported and are inherently subject to information bias. In a validity study of self-reported parental behaviors related to safety practices, sensitivities were high among the parent’s safety practices.^30^ In the current study, parental surveys were completed by parents outside of the context of the checklist program; thus, any misclassification in risk behavior reporting is likely to be independent of checklist implementation. Furthermore, due to the lack of direct injury data of children, we were not able to evaluate the effect of the municipality intervention specifically on injury incidence. Third, this study targeted those who received child health exams at a community health center. While the majority of children received their early health exams at one of these centers, it is possible that certain deprived populations could have been missed. Finally, it cannot be ruled out that the use of the checklist intervention may be a marker for an unobserved municipality characteristic unaccounted for in our analysis, such as other programmatic activities that enhance the social capital environment among parental groups. However, when we examined other types of municipality initiatives implemented for injury prevention (eg, brochures and bulletin board advertisements), there did not appear to be differences in parental behaviors by implementation status of those strategies.

In conclusion, our study showed that a population intervention comprised of using a child safety checklist in the setting of the 4-month infant health exam in Japan may help to promote some child safety parental behaviors, which may translate into a potential reduction in unintentional injuries in children in the future. In this study, we pursued a rigorous approach ensuring that the temporality of the municipal intervention preceded the outcome, but this was at the cost of having to reduce the sample size. Future studies should consider a full longitudinal cohort study approach that allows for the collection of detailed individual-level data over time and the careful adjustment of inter-individual fluctuations when examining the effect of a checklist on the parental risk behaviors.

## APPENDIX A. SUPPLEMENTARY DATA

eAppendix 1. Supplementary materials

eTable 1. Relationship between checklist and the behavior “Tobacco” (tobacco/ashtray out of reach of infant)

eTable 2. Relationship between check list and risk behavior: candy

eTable 3. Relationship between check list and risk behavior: pills/detergents

eTable 4. Relationship between check list and risk behavior: no the child car seat

eTable 5. Relationship between check list and risk behavior: undrained bathwater

eTable 6. Relationship between check list and risk behavior: no lock on Bathing room

eTable 7. Relationship between checklist and parental risk behaviors stratified by birth order and population size of the municipalities

eFigure 1. Checklist to keep your children safe
