# Supplementary material for: Municipality-Level Checklist to Promote Parental Behaviors Related to Prevention of Unintentional Injury in Young Children: A Multilevel Analysis of National Data
Source: J Epidemiol. 2020 Oct 5;30(10):450–6. doi: 10.2188/jea.JE20190079 (PMC7492702; doi:10.2188/jea.JE20190079)
Supplement: Supplementary file 1 [file je-30-450-s001.pdf]

eAppendix 1. Supplementary materials

Municipality-level checklist to promote parental behaviors related to prevention of unintentional injury in young children: a multilevel analysis of national data

METHODS

Healthy Parents and Children 21

The objective of this initiative was to promote a uniform level of maternal and child health services to all children in Japan, including those living in comparatively deprived regions or disadvantaged families. Based on guidelines provided by the Japanese government, each municipality was given the task of implementing measures that addressed issues, such as access to healthcare, support systems continuing from pregnancy, prevention of unintentional injury to children, prevention of child abuse, and assembling informal support systems for parents in the community.

Procedural process for municipality surveys

The municipality surveys were conducted three times; once each in 2005, 2009, and 2013 across all municipalities in Japan. Each municipality government was required by the Ministry of Health, Labor and Welfare to answer the municipality questionnaire for evaluation of "Healthy Parents and Children 21". Questionnaires were sent to each municipality government from the Ministry of Health, Labour and Welfare. Municipality's survey responses were obtained from all municipalities in Japan in each year (response rate was 100% at the municipality level).

Procedural process for parental survey

Unlike the municipality survey, the parental survey was administered in only a subset of municipalities selected randomly after stratifying by population size. The sampling scheme implemented in 2013, the year for which data was used in this current analysis, was pursued as follows. Within each prefecture (large administrative divisions of Japan), municipalities were ranked by ascending population size and were divided into four groups based on quartile cut points. Two municipalities were selected at random from each of the lower two quartile groups, and three were selected from each of the upper two quartile groups, resulting in 10 municipalities per prefecture and 470 totals across Japan (47 prefectures).[1, 2]

The selected municipalities were asked to administer the parental surveys at the timing

of the health exam. All 470 municipalities agreed to both parental survey administration and completed municipality surveys. The parental survey was administered at the time of the child's health exam by the municipality's health care officer at each public facility (e.g., community health center) at 4 months, 1.5 years, and/or 3 years of age. The parental survey was conducted in each municipality from April to August of 2013. Questionnaires were either mailed to the parents prior to the health exam and collected at the time of exam or provided to the parents at the exam and were returned by mail.

We excluded municipalities that did not administer a parental survey at the 1.5-year health exam in 2013 (n=28), and did not report the same checklist implementation status in 2009 and 2013 (n=71). In this study, the parental survey administered at the 1.5-year health examination in 2013 served as the source for obtaining parental behavior data (primary outcome). This meant that the timing of the checklist distribution (primary exposure) should have occurred at the 4-month health examination in 2012. Unfortunately, no municipality survey was conducted in 2012 to directly confirm whether a checklist distribution was implemented. Municipality surveys were conducted only in 2005, 2009, and 2013. In order to ensure the accuracy of the checklist implementation status in 2012 (an interim year), we implemented a criteria in this study that excluded all municipalities in which the reported checklist implementation status was different between the 2009 and 2013 municipality survey results. We pursued the study with the assumption that the checklist implementation status reported in both 2009 and 2013 is the representative status for 2012 as well. For this reason, 71 municipalities (4,528 families) were excluded from our analysis, resulting in the inclusion of 371 municipalities (23,394 families) in the current study.

In addition, health exams at 4 months, 1.5 years, and 3 years old are usually performed at their local public health centers or designated hospitals/clinics administered by the municipality. The municipality invites all families to undergo these health exams where children of the target ages live based on resident registration information. The participating health exam rate is 96%, 96%, 95% for the 4-month, 1.5-year, and 3-year-old health checkups, respectively.[3]

Explanation of covariates

1. Municipality level

We obtained, calculated, and created municipality-level confounding valuables from the "STATISTICAL OBSERVATIONS OF SHI, KU, MACHI, MURA" database published by the Ministry of Internal Affairs and Communications. "Population density" was calculated by dividing the number of people in the population by

habitable area. The variable "0-3 years population rate" was calculated by dividing the population aged 0–3 years by the entire population. "Unemployed rate" was calculated by dividing the total number of unemployed persons by the labor force population and multiplying by 100. "Taxable amount" used the value of municipal-level income in 2013 as a continuous variable.

2. Individual level

2.1 Sex of child: either biological male or female

2.2 Birth order: There were three categories, namely, 1st child, 2nd child, and 3rd child or later. In the sub-analysis (stratify birth order or municipality size), because we thought that the first child had less risk behavior than the second child or later, the categories were changed to 1st child and 2nd child.

2.3 Maternal occupation: For the parental response to the question "Does the mother work now?", the following seven answer choices were available: "full-time", "part-time", "self-employed", "home working", "other", "childcare leave period", or "not employed". Because, except for "other", the mothers were thought to be at home during the day and each sample number was small, we combined "self-employed", "home working", "other", and "childcare leave period" into one category, namely, "self-employed".

2.4 Maternal age: For the parental response to the question "How old were you when you gave birth?", the following six answer choices were available: "19 years old or younger", "20–24 years old", "25–29 years old", "30–34 years old", 35–39 years old or "40 years or older". Because "19 years old or younger" and "40 years old or older" each had sample numbers that were too small, we included "19 years old or younger" in "20–24 years old" and we combined "35–39 years old" and "40 years old or older" into one category, namely, "35 years old or older".

2.5 Self-assessed economic status: For the parental response to the question "How do you feel by comprehensively looking at the current economic situation of daily living?", the following five answer choices were available: "much abundance", "affordable", "standard", "somewhat tight budget", or "very tight budget". Because "much abundance" and "very tight budget" each had sample numbers that were too small, we combined "much abundance" in "affordable" into one category, namely, "high" and we combined "very tight budget" and "somewhat tight budget" into one category, namely, "low".

2.6 Persons to consult: For the parental response to the question "Who is the mother's

counselor for everyday child rearing?”, the following eleven answer choices were available (multiple answers allowed): “discuss with partners”, “grandparents”, “neighbor”, “friends”, “family physician”, “public health nurse and/or midwife”, “nursery teacher or kindergarten teacher”, “telephone consultation”, “the internet”, “others”, and “nobody”. If there was no selection or “nobody” was selected, the response was “no”. If there were one or more selections, the response was “yes”.

2.7 Family physician: For the parental response to the question “Do you have a family physician?”, the following three answer choices were available: “yes”, “no”, or “unclear”.

REFERENCES

1. Zentaro Yamagata, University of Yamanashi research team and the research of Health Labour Sciences Research Grant (#H25-sukoyaka-ippan-002). Report of an study about promotion of Healthy Parents and Children 21. 2016. (in Japanese).
2. Zentaro Yamagata, University of Yamanashi research team and the research of Health Labour Sciences Research Grant (#H24-sukoyaka-ippan-003). Report of an study about promotion of Healthy Parents and Children 21. 2015. (in Japanese).
3. Ministry of Health LaW. Report on Regional Public Health Services and Health Promotion Services; 2016.

eTable 1. Relationship between checklist and the behavior “Tobacco” (tobacco/ashtray out of reach of infant)

	Prevalence of tobacco behavior (%)	Model 0	Model 1 OR (95% CI)	Model 2 OR (95% CI)	Model 3 OR (95% CI)
n (individuals)		13,103	13,103	12,378	12,378
N (municipalities)		370	370	369	369
**[Municipal level]**					
Checklist					0.49 (0.25–0.95)
Population density				1.00 (0.42–0.99)	1.00 (0.99–1.00)
0–3 years population rate				0.77 (0.02–0.63)	0.79 (0.64–0.98)
Unemployment rate				0.96 (0.28–0.89)	0.96 (0.89–1.03)
Taxable income in 2013				1.00 (0.80–1.00)	1.00 (1.00–1.00)
**[Individual level]**					
Maternal age, years					
<24	92 (14.7)		1.19 (0.86–1.66)	1.18 (0.85–1.63)	1.18 (0.85–1.64)
25–29	216 (34.4)		1.19 (0.92–1.54)	1.19 (0.92–1.53)	1.19 (0.93–1.54)
30–34	168 (26.8)		reference	reference	reference
>35	152 (24.2)		1.14 (0.86–1.50)	1.13 (0.85–1.49)	1.14 (0.86–1.50)
Birth order					
First	270 (42.9)		reference	reference	reference
Second	239 (38.0)		1.11 (0.87–1.40)	1.09 (0.86–1.38)	1.09 (0.86–1.38)
Third child or later	120 (19.1)		1.72 (1.31–2.25)	1.69 (0.29–2.22)	1.69 (1.29–2.22)
Child's sex					
Male	313 (50.0)		reference	reference	reference
Female	313 (50.0)		0.97 (0.79–1.18)	0.97 (0.79–1.18)	0.97 (0.80–1.18)
Maternal occupation					
Not employed	292 (48.0)		reference	reference	reference
Full-time	100 (16.4)		1.42 (1.10–1.84)	1.39 (1.07–1.79)	1.38 (1.07–1.78)
Part-time	133 (21.8)		1.26 (0.96–1.65)	1.24 (0.94–1.62)	1.24 (0.94–1.62)
Self-employed or others	84 (13.8)		1.4 (1.01–1.93)	1.37 (0.99–1.89)	1.37 (0.99–1.90)
Self-assessed economic status					
High	57 (9.3)		1.01 (0.70–1.45)	0.99 (0.69–1.43)	0.99 (0.69–1.43)
Standard	323 (52.9)		reference	reference	reference
Low	231 (37.8)		1.41 (1.14–1.74)	1.42 (1.15–1.75)	1.42 (1.15–1.76)
Persons to consult					
Yes	608 (96.7)		reference	reference	reference
No	21 (3.3)		1.54 (0.47–5.02)	1.54 (0.47–5.04)	1.53 (0.47–4.99)
Have family physician					
Yes	553 (90.8)		reference	reference	reference
No	19 (3.1)		1.7 (1.04–2.79)	1.69 (1.03–2.78)	1.70 (1.03–2.78)

Unclear	37 (6.1)		1.69 (1.16–2.47)	1.62 (1.11–2.38)	1.63 (1.11–2.38)
**[Random effect]**					
Variance between municipalities		0.17	0.16	0.14	0.13
Proportional change in the variance[a]		reference	6%	18	24%

CI, confidence interval; n, number of participants; OR, odds ratio.

[a] PCV = {τ00 (a) − τ00 (b) / τ00 (a)} × 100 = xx%

eTable 2. Relationship between check list and risk behavior: candy

	Prevalence of candy behavior (%)	Model 0	Model 1 OR (95% CI)	Model 2 OR (95% CI)	Model 3 OR (95% CI)
n (individuals)		22,769	21,647	21,610	21,610
N (municipalities)		371	371	370	370
**[Municipal level]**					
Checklist					0.54 (0.39–0.75)
Population density				1.00 (1.00–1.00)	1.00 (1.00–1.00)
0–3 years population rate				0.89 (0.80–0.99)	0.91 (0.82–1.01)
Unemployment rate				0.96 (0.93–1.00)	0.96 (0.93–1.00)
Taxable income in 2013				1.00 (1.00–1.00)	1.00 (1.00–1.00)
**[Individual level]**					
Maternal age, years					
<24	126 (11.4)		0.93 (0.76–1.15)	0.94 (0.76–1.16)	0.94 (0.76–1.16)
25–29	348 (31.4)		0.94 (0.82–1.08)	0.95 (0.82–1.09)	0.95 (0.82–1.09)
30–34	345 (31.2)		reference	reference	reference
>35	288 (26.0)		0.97 (0.84–1.11)	0.97 (0.84–1.11)	0.97 (0.85–1.12)
Birth order					
First	493 (44.5)		reference	reference	reference
Second	418 (37.7)		1.8 (1.57–2.05)	1.80 (1.57–2.06)	1.80 (1.58–2.06)
Third child or later	197 (17.8)		3.07 (2.64–3.58)	3.10 (2.66–3.61)	3.11 (2.67–3.62)
Child's sex					
Male	558 (50.5)		reference	reference	reference
Female	546 (49.5)		1.04 (0.93–1.15)	1.04 (0.93–1.16)	1.04 (0.93–1.16)
Maternal occupation					
Not employed	559 (52.1)		reference	reference	reference
Full-time	182 (17.0)		1.49 (0.31–1.71)	1.49 (1.30–1.70)	1.48 (1.29–1.69)
Part-time	198 (18.4)		1.08 (0.93–1.26)	1.08 (0.93–1.27)	1.08 (0.93–1.26)
Self-employed or others	135 (12.6)		1.08 (0.90–1.31)	1.08 (0.89–1.30)	1.08 (0.89–1.30)
Self-assessed economic status					
High	123 (11.5)		0.98 (0.82–1.17)	0.97 (0.81–1.16)	0.97 (0.81–1.17)
Standard	600 (55.9)		reference	reference	reference
Low	351 (32.7)		1.07 (0.95–1.21)	1.07 (0.95–1.21)	1.07 (0.95–1.21)
Persons to consult					
Yes	1073 (96.8)		reference	reference	reference
No	35 (3.2)		1.89 (1.02–3.49)	1.87 (1.01–3.45)	1.85 (1.00–3.42)
Have family physician					
Yes	965 (89.9)		reference	reference	reference
No	33 (3.1)		1.38 (1.01–1.88)	1.37 (1.00–1.87)	1.38 (1.01–1.88)

Unclear	76 (7.1)		1.49 (1.20–1.86)	1.49 (1.20–1.86)	1.50 (1.21–1.87)
**[Random effect]**					
Variance between municipalities		0.02	0.01	0.00	0.00
Proportional change in the variance[a]		reference	50%	100%	100%

CI, confidence interval; n, number of participants; OR, odds ratio.

[a] PCV = $\{\tau 00\ (a) - \tau 00\ (b) / \tau 00\ (a)\} \times 100$ = xx%

eTable 3. Relationship between check list and risk behavior: pills/detergents

	Prevalence of pills/detergents behavior (%)	Model 0	Model 1 OR (95% CI)	Model 2 OR (95% CI)	Model 3 OR (95% CI)
n (individuals)		22,769	21,648	21,611	21,611
N (municipalities)		371	371	370	370
**[Municipal level]**					
Checklist					0.89 (0.72–1.09)
Population density				1.00 (1.00–1.00)	1.00 (1.00–1.00)
0–3 years population rate				1.02 (0.94–1.11)	1.03 (0.95–1.11)
Unemployment rate				0.96 (0.93–0.98)	0.96 (0.93–0.98)
Taxable income in 2013				1.00 (1.00–1.00)	1.00 (1.00–1.00)
**[Individual level]**					
Maternal age, years					
<24	125 (11.3)		0.65 (0.57–0.74)	0.66 (0.58–0.75)	0.66 (0.58–0.75)
25–29	349 (31.4)		0.93 (0.85–1.01)	0.93 (0.85–1.02)	0.93 (0.85–1.02)
30–34	346 (31.2)		reference	reference	reference
>35	290 (26.1)		1.03 (0.94–1.13)	1.03 (0.94–1.12)	1.03 (0.94–1.12)
Birth order					
First	492 (44.3)		reference	reference	reference
Second	420 (37.8)		0.90 (0.83–0.97)	0.90 (0.83–0.97)	0.90 (0.83–0.97)
Third child or later	199 (17.9)		0.90 (0.81–1.00)	0.91 (0.82–1.01)	0.91 (0.82–1.01)
Child's sex					
Male	559 (50.5)		reference	reference	reference
Female	548 (49.5)		1.12 (1.04–1.20)	1.12 (1.04–1.20)	1.12 (1.04–1.20)
Maternal occupation					
Not employed	559 (51.9)		reference	reference	reference
Full-time	183 (17.0)		1.48 (1.36–1.61)	1.49 (1.37–1.63)	1.49 (1.37–1.62)
Part-time	199 (18.5)		1.15 (1.04–1.27)	1.15 (1.04–1.28)	1.15 (1.04–1.28)
Self-employed or others	136 (12.6)		1.21 (1.08–1.36)	1.21 (1.08–1.36)	1.21 (1.08–1.36)
Self-assessed economic status					
High	123 (11.4)		0.94 (0.84–1.05)	0.94 (0.84–1.05)	0.94 (0.84–1.05)
Standard	601 (55.8)		reference	reference	reference
Low	353 (32.8)		1.17 (1.08–1.26)	1.17 (1.08–1.26)	1.17 (1.08–1.26)
Persons to consult					
Yes	1076 (96.9)		reference	reference	reference
No	35 (3.2)		0.96 (0.57–1.61)	0.95 (0.56–1.59)	0.94 (0.56–1.58)
Have family physician					
Yes	968 (89.9)		reference	reference	reference

No	33 (3.1)		1.12 (0.91–1.37)	1.12 (0.91–1.38)	1.12 (0.91–1.38)
Unclear	76 (7.1)		1.47 (1.28–1.69)	1.47 (1.28–1.69)	1.47 (1.28–1.69)
**[Random effect]**					
Variance between municipalities		0.06	0.05	0.05	0.04
Proportional change in the variance[a]		ref reference	17%	17%	33%

CI, confidence interval; n, number of participants; OR, odds ratio.

[a] PCV = $\{\tau 00\ (a) - \tau 00\ (b) / \tau 00\ (a)\} \times 100$ = xx%

eTable 4. Relationship between check list and risk behavior: no the child car seat

n (individuals)<br>N (municipalities)	Prevalence of no child car seat behavior (%)	Model 0	Model 1 OR (95% CI)	Model 2 OR (95% CI)	Model 3 OR (95% CI)
n (individuals)		21,340	20,296	20,259	20,259
N (municipalities)		371	371	370	370
**[Municipal level]**					
Checklist					0.72 (0.54–0.97)
Population density				1.00 (0.99–1.00)	1.00 (0.99–1.00)
0-3 years population rate				0.96 (0.86–1.06)	0.97 (0.87–1.07)
Unemployment rate				1.08 (1.05–1.12)	1.08 (1.05–1.12)
Taxable income 2013year				1.00 (1.00–1.00)	1.00 (1.00–1.00)
**[Individual level]**					
Maternal age, years					
<24	117 (11.1)		20.9 (1.80–2.41)	2.03 (1.75–2.34)	2.03 (1.76–2.35)
2–29	330 (31.3)		1.25 (1.11–1.41)	1.24 (1.10–1.39)	1.24 (1.10–1.39)
30–34	326 (31.0)		reference	reference	reference
>35	280 (26.6)		0.95 (0.83–1.08)	0.95 (0.83–1.08)	0.95 (0.83–1.08)
Birth order					
First	465 (44.1)		reference	reference	reference
Second	402 (38.1)		0.74 (0.66–0.83)	0.73 (0.66–0.82)	0.73 (0.66–0.82)
Third child or later	187 (17.7)		1.47 (1.30–1.67)	1.45 (1.27–1.64)	1.45 (1.27–1.64)
Child's sex					
Male	525 (50.0)		reference	reference	reference
Female	525 (50.0)		1.04 (0.95–1.14)	1.04 (0.95–1.14)	1.04 (0.95–1.14)
Maternal occupation					
Not employed	533 (52.1)		reference	reference	reference
Full-time	178 (17.4)		1.20 (1.07–1.36)	1.19 (1.06–1.34)	1.19 (1.06–1.34)
Part-time	189 (18.5)		1.17 (1.03–1.32)	1.15 (1.02–1.31)	1.15 (1.01–1.31)
Self-employed or others	123 (12.0)		1.25 (1.07–1.46)	1.24 (1.07–1.45)	1.24 (1.07–1.45)
Self-assessed economic status					
High	119 (11.6)		1.01 (0.86–1.18)	1.03 (0.88–1.20)	1.03 (0.88–1.20)
Standard	571 (55.9)		reference	reference	reference
Low	332 (32.5)		1.41 (1.28–1.56)	1.41 (1.27–1.55)	1.41 (1.27–1.55)
Persons to consult					
Yes	1021 (96.9)		reference	reference	reference
No	33 (3.1)		1.28 (0.69–2.39)	1.30 (0.70–2.42)	1.29 (0.69–2.41)
Have family physician					
Yes	916 (89.5)		reference	reference	reference

No	31 (3.0)		1.09 (0.84–1.43)	1.09 (0.83–1.43)	1.09 (0.83–1.43)
Unclear	76 (7.4)		1.12 (0.92–1.36)	1.12 (0.92–1.37)	1.13 (0.92–1.37)
**[Random effect]**					
Variance between municipalities		0.16	0.12	0.09	0.08
Proportional change in the variance[a]		reference	25%	44%	50%

CI, confidence interval; n, number of participants; OR, odds ratio.

[a] PCV = {τ00 (a) − τ00 (b) / τ00 (a)} × 100 = xx%

eTable 5. Relationship between check list and risk behavior: undrained bathwater

	Prevalence of undrained bathwater behavior (%)	Model 0	Model 1 OR (95% CI)	Model 2 OR (95% CI)	Model 3 OR (95% CI)
n (individuals)		22,143	21,069	21,032	21,032
N (municipalities)		371	371	370	370
**[Municipal level]**					
Checklist					0.90 (0.67–1.21)
Population density				1.00 (1.00–1.01)	1.00 (1.00–1.01)
0–3 years population rate				0.90 (0.81–1.01)	0.90 (0.81–1.01)
Unemployment rate				0.97 (0.93–1.01)	0.97 (0.93–1.01)
Taxable income in 2013				1.00 (1.00–1.00)	1.00 (1.00–1.00)
**[Individual level]**					
Maternal age, years					
<24	123 (11.4)		0.76 (0.67–0.86)	0.77 (0.68–0.86)	0.77 (0.68–0.86)
25–29	338 (31.2)		0.96 (0.89–1.04)	0.97 (0.89–1.05)	0.97 (0.89–1.05)
30–34	342 (31.6)		reference	reference	reference
>35	281 (25.9)		1.00 (0.92–1.09)	1.00 (0.92–1.09)	1.00 (0.92–1.09)
Birth order					
First	480 (44.2)		reference	reference	reference
Second	410 (37.8)		1.12 (1.04–1.20)	1.12 (1.05–1.21)	1.12 (1.05–1.21)
Third child or later	195 (18.0)		1.21 (1.10–1.33)	1.23 (1.11–1.35)	1.23 (1.11–1.35)
Child's sex					
Male	545 (50.4)		reference	reference	reference
Female	537 (49.6)		1.02 (0.95–1.08)	1.02 (0.95–1.09)	1.02 (0.95–1.09)
Maternal occupation					
Not employed	549 (52.1)		reference	reference	reference
Full-time	178 (16.9)		1.16 (1.07–1.26)	1.16 (1.07–1.26)	1.16 (1.07–1.26)
Part-time	193 (18.3)		1.04 (0.94–1.14)	1.04 (0.94–1.14)	1.04 (0.94–1.14)
Self-employed or others	133 (12.6)		1.01 (0.90–1.13)	1.01 (0.91–1.13)	1.01 (0.91–1.13)
Self-assessed economic status					
High	122 (11.6)		0.95 (0.85–1.05)	0.94 (0.85–1.05)	0.94 (0.85–1.05)
Standard	587 (55.8)		reference	reference	reference
Low	344 (32.7)		1.34 (1.25–1.44)	1.34 (1.25–1.44)	1.34 (1.25–1.44)
Persons to consult					
Yes	1053 (97.0)		reference	reference	reference
No	32 (3.0)		1.56 (1.00–2.43)	1.55 (0.99–2.42)	1.55 (0.99–2.42)
Have family physician					

Yes	949 (90.0)		reference	reference	reference
No	33 (3.1)		1.22 (1.01–1.48)	1.22 (1.01–1.47)	1.22 (1.01–1.47)
Unclear	73 (6.9)		1.23 (1.07–1.41)	1.24 (1.08–1.42)	1.24 (1.08–1.42)
**[Random effect]**					
Variance between municipalities		*0.25*	0.25	0.22	0.22
Proportional change in the variance[a]		reference	0%	12%	12%

CI, confidence interval; n, number of participants; OR, odds ratio.

[a] PCV = {τ00 (a) − τ00 (b) / τ00 (a)} × 100 = xx%

eTable 6. Relationship between check list and risk behavior: no lock on Bathing room

		Model 0	Model 1 OR (95% CI)	Model 2 OR (95% CI)	Model 3 OR (95% CI)
n (individuals)	Prevalence of no lock on bathing room behavior	22,239	21,153	21,117	21,117
N (municipalities)	(%)	371	371	370	370
**[Municipal level]**					
Checklist					0.85 (0.73–0.99)
Population density				1.00 (1.00–1.00)	1.00 (1.00–1.00)
0–3 years population rate				0.95 (0.89–1.01)	0.95 (0.90–1.01)
Unemployment rate				0.99 (0.97–1.01)	0.99 (0.97–1.01)
Taxable income in 2013				1.00 (1.00–1.00)	1.00 (1.00–1.00)
**[Individual level]**					
Maternal age, years					
<24	125 (11.5)		0.93 (0.85–1.03)	0.94 (0.85–1.04)	0.94 (0.85–1.04)
25–29	337 (31.0)		1.00 (0.93–1.07)	1.00 (0.94–1.08)	1.01 (0.94–1.08)
30–34	339 (31.2)		reference	reference	reference
>35	286 (26.3)		0.98 (0.91–1.06)	0.98 (0.91–1.06)	0.98 (0.91–1.06)
Birth order					
First	482 (44.3)		reference	reference	reference
Second	411 (37.8)		1.00 (0.94–1.06)	1.00 (0.94–1.07)	1.00 (0.94–1.07)
Third child or later	195 (17.9)		1.01 (0.93–1.10)	1.02 (0.93–1.11)	1.02 (0.93–1.11)
Child's sex					
Male	550 (50.7)		reference	reference	reference
Female	534 (49.3)		1.10 (1.04–1.16)	1.10 (1.04–1.16)	1.10 (1.04–1.16)
Maternal occupation					
Not employed	548 (51.9)		reference	reference	reference
Full-time	179 (17.0)		1.13 (1.05–1.21)	1.13 (1.05–1.21)	1.12 (1.05–1.21)
Part-time	193 (18.3)		1.02 (0.94–1.10)	1.02 (0.94–1.11)	1.02 (0.94–1.11)
Self-employed or others	136 (12.9)		0.98 (0.90–1.08)	0.99 (0.90–1.08)	0.99 (0.90–1.09)
Self-assessed economic status					
High	118 (11.2)		0.88 (0.81–0.96)	0.87 (0.80–0.96)	0.87 (0.80–0.95)
Standard	591 (56.0)		reference	reference	reference
Low	346 (32.8)		1.18 (1.11–1.26)	1.18 (1.11–1.26)	1.18 (1.11–1.26)
Persons to consult					
Yes	1054 (96.9)		reference	reference	reference

No	34 (3.1)		0.98 (0.65–1.48)	0.98 (0.64–1.48)	0.97 (0.64–1.47)
Have family physician					
Yes	949 (89.9)		reference	reference	reference
No	32 (3.0)		1.39 (1.16–1.65)	1.38 (1.16–1.64)	1.38 (1.16–1.64)
Unclear	75 (7.1)		1.40 (1.23–1.60)	1.40 (1.23–1.60)	1.41 (1.23–1.60)
**[Random effect]**					
Variance between municipalities		*0.03*	0.02	0.02	0.02
Proportional change in the variance[a]		reference	33%	33%	33%

CI, confidence interval; n, number of participants; OR, odds ratio.

[a] PCV = {τ00 (a) − τ00 (b) / τ00 (a)} × 100 = xx%

eTable 7. Relationship between checklist and parental risk behaviors stratified by birth order and population size of the municipalities

	Birth order						Population size[b]		
	First child	Second child	Third or higher	p for interaction; First vs Second[a]	p for interaction; First vs Third or higher[a]	p for interaction; Second vs Third or higher[a]	Small-Medium	Medium-Large	p for interaction[a]
	OR (95% CI)	OR (95% CI)	OR (95% CI)				OR (95% CI)	OR (95% CI)	
Tobacco	0.45 (0.17–1.25)	0.29 (0.07–1.20)	0.75 (0.27–2.11)	0.53	0.43	<0.01	0.84 (0.24–3.01)	0.42 (0.19–0.93)	0.73
Candy	0.40 (0.20–0.82)	0.69 (0.43–1.10)	0.49 (0.27–0.90)	0.12	0.60	0.35	0.56 (0.28–1.14)	0.56 (0.38–0.80)	0.85
No child car seat	0.77 (0.53–1.12)	0.69 (0.44–1.10)	0.67 (0.39–1.14)	0.69	0.51	0.93	0.61 (0.36–1.04)	0.80 (0.57–1.14)	0.38
No lock on bathing room	0.90 (0.74–1.10)	0.86 (0.68–1.09)	0.72 (0.53–0.97)	0.74	0.33	0.24	0.76 (0.57–1.00)	0.90 (0.75–1.08)	0.46

CI, confidence interval; OR, odds ratio.

[a] p values were estimated based on a multilevel model that included a term representing the multiplicative interaction between checklist status and birth order (or population size).

[b] Population sizes: using the parental data-sampling segment of 2013. In this study, a small-to-medium–sized population was defined as 0% to 50% interquartile range of the segment groups, and a medium-to-large–sized population was defined as 50% to 100% interquartile range of those groups.

eFigure 1. Checklist to keep your children safe

From 3 months old child to 1.5 years old child-

Are you doing the following behavior to prevent unintentional injuries to child?

1	You keep a baby bed fence installed	yes	no
2	You do not leave your baby alone on the sofa	yes	no
3	Fences for preventing your baby from falling are installed on the upper and lower floors of the stairs	yes	no
4	You cover the sharp corners of tables with cushions	yes	no
5	Your child's chair is stable	yes	no
6	You always keep tobacco or ashtrays out of your child's reach	yes	no
7	You always keep small objects (such as buttons, candy, pills/detergents) out of your child's reach	yes	no
8	You always keep plastic bags out of your child's reach	yes	no
9	After breastfeeding or bottle-feeding you always burp your baby before laying your baby down	yes	no
10	You always keep pots and rice cooker, which can cause burns, out of your child's reach	yes	no
11	You always keep hot tea, miso soup, cup noodles, etc. in the center of the table where out of reach of children	yes	no
12	You do not use any table cloth	yes	no
13	After you use an iron, cool it out of the reach of children	yes	no
14	You install a fence around the stove and heaters	yes	no
15	You keep guard so that children's fingers will not enter the hinged part of a door	yes	no
16	You keep your child's fingers from getting into a glass door or video tape deck	yes	no
17	After you use an edge tool (such as a razor, scissors, or knife), you immediately return it to a locked drawer	yes	no
18	You do not leave your baby in the bathing room alone, and after taking a bath, you drain the hot water in the bathtub	yes	no
19	You keep the door locked so that your baby does not enter into in the bath alone	yes	no
20	You installed a child car seat in the back seat	yes	no

If you selected "No", read the leaflet and take appropriate behavior to prevent unintentional injuries to your child. (Created by T. Tanaka)

The guidelines have not been revised at least last 10 years.
